# Evaluating the Targeting of a *Staphylococcus-aureus*-Infected Implant with a Radiolabeled Antibody In Vivo

**DOI:** 10.3390/ijms24054374

**Published:** 2023-02-22

**Authors:** Bruce van Dijk, J. Fred F. Hooning van Duyvenbode, Lisanne de Vor, F. Ruben H. A. Nurmohamed, Marnix G. E. H. Lam, Alex J. Poot, Ruud M. Ramakers, Sofia Koustoulidou, Freek J. Beekman, Jos van Strijp, Suzan H. M. Rooijakkers, Ekaterina Dadachova, H. Charles Vogely, Harrie Weinans, Bart C. H. van der Wal

**Affiliations:** 1 Department of Orthopedics, University Medical Center Utrecht, 3584 CX Utrecht, The Netherlands; 2 Department of Medical Microbiology, University Medical Centre Utrecht, 3584 CX Utrecht, The Netherlands; 3 Department of Radiology and Nuclear Medicine, University Medical Center Utrecht, 3584 CX Utrecht, The Netherlands; 4 MILabs B.V., 3584 CX Utrecht, The Netherlands; 5 Department of Radiation Science and Technology, Delft University of Technology, 2628 CD Delft, The Netherlands; 6 Department of Translational Neuroscience, Brain Center Rudolf Magnus, University Medical Center, 3584 CX Utrecht, The Netherlands; 7 College of Pharmacy and Nutrition, University of Saskatchewan, Saskatoon, SK S7N 5A8, Canada; 8 Department of BioMechanical Engineering, Delft University of Technology, 2628 CD Delft, The Netherlands

**Keywords:** antibody, biodistribution, infection, mice, periprosthetic joint infection, radioimmunotherapy, theranostics, radiolabeling, *S. aureus*, SPECT

## Abstract

Implant infections caused by *Staphylococcus aureus* are difficult to treat due to biofilm formation, which complicates surgical and antibiotic treatment. We introduce an alternative approach using monoclonal antibodies (mAbs) targeting *S. aureus* and provide evidence of the specificity and biodistribution of *S.*-*aureus*-targeting antibodies in a mouse implant infection model. The monoclonal antibody 4497-IgG1 targeting wall teichoic acid in *S. aureus* was labeled with indium-111 using CHX-A”-DTPA as a chelator. Single Photon Emission Computed Tomography/computed tomographyscans were performed at 24, 72 and 120 h after administration of the ^111^In-4497 mAb in Balb/cAnNCrl mice with a subcutaneous implant that was pre-colonized with *S. aureus* biofilm. The biodistribution of this labelled antibody over various organs was visualized and quantified using SPECT/CT imaging, and was compared to the uptake at the target tissue with the implanted infection. Uptake of the ^111^In-4497 mAbs at the infected implant gradually increased from 8.34 %ID/cm^3^ at 24 h to 9.22 %ID/cm^3^ at 120 h. Uptake at the heart/blood pool decreased over time from 11.60 to 7.58 %ID/cm^3^, whereas the uptake in the other organs decreased from 7.26 to less than 4.66 %ID/cm^3^ at 120 h. The effective half-life of ^111^In-4497 mAbs was determined to be 59 h. In conclusion, ^111^In-4497 mAbs were found to specifically detect *S. aureus* and its biofilm with excellent and prolonged accumulation at the site of the colonized implant. Therefore, it has the potential to serve as a drug delivery system for the diagnostic and bactericidal treatment of biofilm.

## 1. Introduction

Healthcare-associated infections caused by *Staphylococcus aureus* are responsible for high morbidity and mortality, especially after medical procedures involving prosthetic implants [1,2]. These infections are difficult to treat due to biofilm formation on the prosthetic material [3]. As a physical barrier, biofilms hinder the host immune system [4,5] and can also prevent antibiotics from reaching the bacteria, thus increasing antibiotic resistance. In addition, the bacteria in a biofilm are mostly in a metabolically inactive state and therefore are not susceptible to most antibiotics [6]. These metabolically inactive bacteria can become active again, causing reinfection and potentially increasing antibiotic resistance even further. The treatment of (peri)prosthetic joint infection often involves the long-term use of antibiotics and surgery with or without removal of the implant. Despite this intensive treatment, the outcome is still unpredictable. In addition, older patients with prosthetic joint infection usually have multiple comorbidities, which requires multimodal treatment. Therefore, these patients bear resemblance to oncology patients, with comparably high morbidity and mortality rates. The 5-year mortality of prosthetic joint infections is even higher than that of most forms of breast, prostate and thyroid cancer [7,8]. Consequently, alternative treatment options need to be explored, and knowledge on therapies applied in oncology could potentially be used to treat prosthetic infections.

Monoclonal antibodies (mAbs), as carriers for radiodiagnostic or radioimmunotherapeutic (RIT) isotopes, may provide an alternative approach to improve the diagnosis and treatment of *S. aureus* biofilm-related infections. Recent developments have seen the resurgent role of mAbs in the diagnosis of invasive fungal infections in patients, as well as in localizing HIV reservoirs in HIV-infected individuals [9,10]. Radioimmunotherapy is used to treat multiple types of cancer and relies on the antigen-binding characteristics of the mAbs to deliver cytotoxic radiation to target malignant cells [11]. Antibodies have been proposed as delivery vehicles for the radioimmunotherapy of infectious diseases [12], and a recent review highlights the multiple pre-clinical applications of RIT for therapy for various classes of infectious agents [13]. Theranostics is an emerging field in oncology that combines molecular imaging and specific targeted therapy in the same agent. When combined, non-invasive molecular imaging techniques, such as Single Photon Emission Computed Tomography (SPECT), could potentially elucidate the whole-body distribution of radiolabeled mAbs uptake in relation to the infected area and could predict its bactericidal effect. The key to a successful theranostic approach is a specific vehicle, e.g., proteins, nanobodies or peptides with high affinity and selectivity for the target cells. Furthermore, biodistribution and pharmacokinetics are fundamental aspects of understanding and predicting the efficacy and toxicity of potential theranostic agents.

The monoclonal antibody 4497-IgG1 (anti-β-GlcNAc WTA) specifically recognizes clinically relevant *S. aureus* biofilm types in vitro and targets *S. aureus* biofilm in vivo [14]. The antibody 4497-IgG1 targets wall teichoic acids (WTA) [15,16], which are found in both the bacterial cell wall and within the extracellular matrix of the biofilm, making it an ideal carrier for antibacterial and biofilm agents, such as enzymes, photosensitizers or therapeutic radionuclides against *S. aureus* biofilms. For example, previous in vitro results showed that this antibody charged with alpha-radiation emitting Bismuth-213 can selectively kill *S. aureus* cells in vitro in both planktonic and biofilm states [17]. The next step in the pre-clinical development of this potential radiodiagnostic and therapeutic *S. aureus* targeting antibody is determining its biodistribution throughout other organs. The aim of this study was to analyze the biodistribution and the whole-body clearance of 4497-IgG1 antibodies in a subcutaneous implant infection in mice. The antibody was radiolabeled with Indium-111 (^111^In), after which visualization and quantification was performed using software analyses on SPECT/computed tomography (CT) images.

## 2. Results

**Figure 1:**
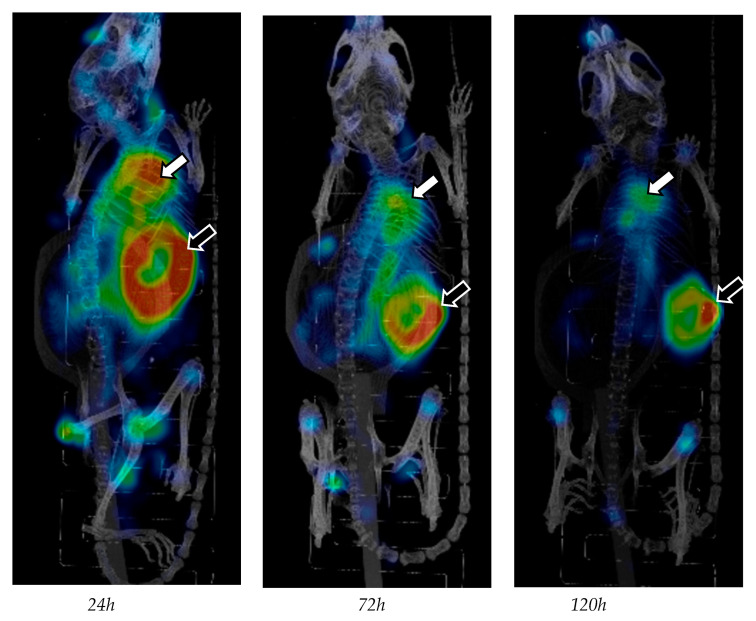
Reconstructed 3D body scans were visualized using maximum intensity projection at 24, 72 and 120 h, and the SPECT scale was adjusted by cutting 10% of the lower signal intensity to make the high-intensity regions readily visible. The white arrow points to the heart and the black arrow points to the infected implant. An increased signal is seen at the hips and knees probably due to detached ^111^In.

**Figure 2:**
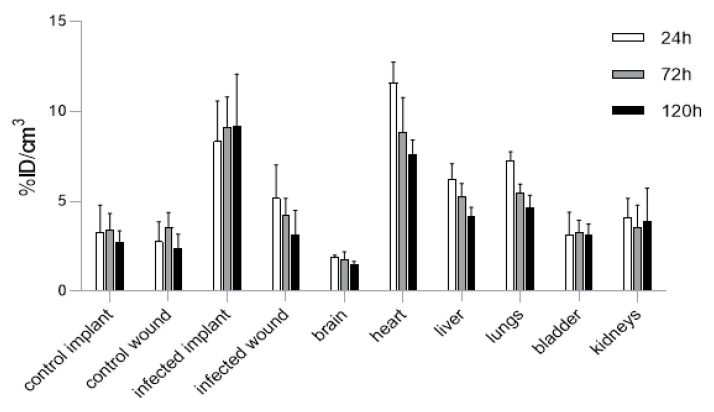
Biodistribution of ^111^In-labeled 4497 antibody targeting *S. aureus* in seven BalB/C mice with a subcutaneous implant infection at multiple timepoints [14]. The bars represent 24, 72 and 120 h after IV administration. At 72 and 120 h, significantly higher uptake was seen at the implant infection site and the heart compared to all the other organs or targeted areas (*p* ≤ 0.002).

The biodistribution of 4497-^111^In was quantified in seven mice at 24, 72 and 120 h. The maximum intensity projections of the SPECT/CT scans showed increased uptake at the infected implant and heart (Figure 1). As shown previously [14], 4497 accumulated specifically and continuously at the implant infection site. The uptake was 8.34 ± 2.25 %ID/cm^3^ around 24 h post-injection, after which the signal gradually increased to 9.15 ± 1.67 %ID/cm^3^ and 9.22 ± 2.86 %ID/cm^3^ at 72 h and 120 h, respectively. At all the timepoints, there was a significantly higher accumulation at the implant infection compared to the control implant in each mouse, with 3.9 ± 1.51 %ID/cm^3^ (24 h), 3.43 ± 0.91 %ID/cm^3^ (72 h) and 2.73 ± 0.64 %ID/cm^3^ (120 h). When comparing the uptake in the organs and targeted areas, statistical analyses showed a significantly higher uptake (11.60 ± 1.16%) in the heart compared to the other organs and the infected implant (*p* ≤ 0.01) at 24 h. Additionally, uptake at the infected implant at 24 h was significantly higher compared to the other organs except for the heart, liver and lungs. There was no significant difference between the uptake in the liver (6.25 ± 0.86 %ID/cm^3^, *p* = 0.125) and lungs (7.26 ± 0.51 %ID/cm^3^, *p* = 1.000) compared to the implant infection site. At 72 and 120 h, significantly higher uptake was seen at the implant infection site compared to all the other organs or targeted areas (*p* ≤ 0.001), except for the heart with 8.86 ± 1.92 %ID/cm^3^, which showed no significant reduction relative to the target infection site (*p* = 1.000) with 7.58 ± 0.84 %ID/cm^3^ (*p* = 0.960), as shown in Figure 2. Additionally, the ratio between the infected implant and the blood pool (i.e., muscle) was 4.79:1 at 24 h, 5.35:1 at 72 h and 5.78:1 at 120 h. The data of individual mice and the results of the statistical analyses are available in the supporting information file.

**Figure 3:**
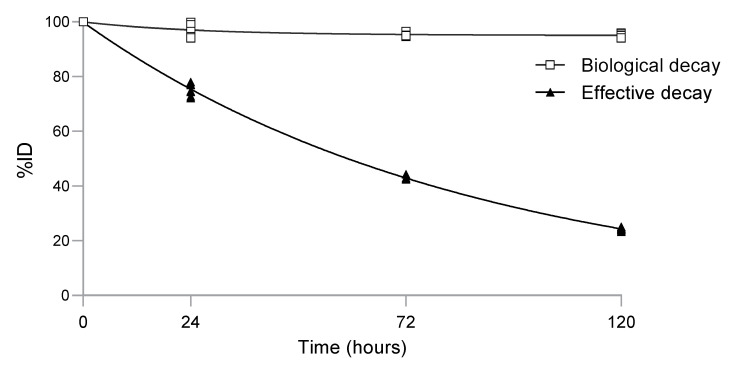
Whole body clearance of 4497-^111^In in seven mice. Curve fitting using nonlinear regression analyses (r^2^ = 0.998) showed that the effective half-life of 4497-^111^In was 59 h.

The activity in each mouse decreased over time. This decrease was due to the effective half-life of 4497-^111^In. The analyses showed an effective half-life of 59 h (Figure 3) for 4497-IgG1-CHX-A”-^111^In. The physical half-life of ^111^In is 67 h, resulting in a decay of 0.78 after 24 h, 0.48 after 72 h and 0.29 after 120 h after the time of injection. The biological half-life was calculated to be 522 h.

The biological clearance as a percentage of the injected dose was 2.97 ± 2.23, 4.56 ± 0.79 and 4.92 ± 0.59% at 24, 72 and 120 h, respectively. The biological decay reached a plateau phase between 24 and 72 h.

## 3. Materials and Methods

### 3.1. S. aureus Targeting Antibodies and Radiolabeling

The 4497-IgG1 (anti-β-GlcNAc WTA) antibodies were synthesized as described before [14], after which the antibodies were conjugated to the bifunctional chelator CHX-A”-DTPA before labeling with ^111^In. CHX-A”-DTPA was chosen because it is a semi-rigid chelator that provides stable labeling at an ambient temperature [18]. The labeling of the antibodies with ^111^In was performed as described previously [14]. In short, the antibodies were incubated at 37 °C for 1.5 h with a 5-fold molar excess of bifunctional CHX-A”-DTPA (Macrocyclics, Plano, TX, USA) in a conjugation buffer. In order to remove the unbound CHX-A”-DTPA, a mAb-CHX-A”-DTPA conjugate was exchanged into an ammonium acetate buffer using Amicon filters (Millipore, Burlington, MA, USA). The antibody conjugates were prepared less than 24 h before use. Radiolabeling with ^111^In was performed in order to achieve a specific antibody activity of 150 kBq/μg. The reaction mixture was incubated for 60 min at 37 °C, after which free ^111^In was bound by quenching the reaction with EDTA solution. The radiolabeling yield was measured by instant thin layer chromatography (iTLC) and confirmed the radiolabeling of at least 95% of the antibodies without the need for further purification.

### 3.2. Biofilm Culture and Subcutaneous Implant Infection Mouse Model

A group of 10 Balb/cAnNCrl male mice weighing >20 g obtained from Charles River Laboratories were used in this experiment. Each mouse received a biofilm infected implant in one flank and a sterile control in the other flank (the left and right side was randomized) as described previously [14]. In short, the implants were made by cutting a 5 mm segment of a 7 French polyurethane catheter (Access Technologies, Niles, MI, USA). The infected implants were pre-colonized with the biofilm of a luminescent strain of methicillin-resistant *S. aureus*, USA300 LAC (AH4802) [19]. An inoculum of ~10^7^ CFU was used, after which the biofilms were grown for 48 h before implantation. The implantation was performed carefully using a 14-gauge guiding needle through which the catheter implants could be positioned correctly using a K-wire. The implantation of colonized and sterile implants was randomized to the left or right flank of the mouse. Seven mice were successfully injected intravenously in the tail with 50 µg of radiolabeled antibody (7.5 MBq) two days after subcutaneous implantation. Incorrect injections were determined by SPECT/CT scans at 24 h. Three mice showed high uptake (>25% of total activity) in the tail, and thus were injected intramuscularly and were therefore excluded.

### 3.3. Biodistribution Quantification and Visualization Using SPECT-CT

The accumulation of radiolabeled antibody was visualized using multimodality SPECT/CT imaging (VECTor^6^ CT scanner, MILabs B.V., Houten, The Netherlands). The imaging of mice was performed at 24, 72 and 120 h post-injection using a mouse collimator (HE-UHR-M) with 162 pinholes (diameter 0.75 mm). The scanning time at 24 h was 30 min. Subsequently, scanning time was corrected for the decay of ^111^In at 72 h (a 42 min scan time) and 120 h (a 60 min scan time). All the mice were sacrificed immediately after the last scan by cervical dislocation while under general anesthesia. SPECT image reconstruction was performed using Similarity Regulated OSEM [20] with 6 iterations and 128 subsets. PMOD Version 4.103 (PMOD Technologies Ltd., Zurich, Switzerland) was used for image processing and the volume of interest analysis. The SPECT scans were individually corrected for the decay of ^111^In at each timepoint and were smoothed using a 1.5 mm 3D gaussian filter. The biodistribution of the 4497-^111^In was analyzed by quantifying the accumulation of ^111^In in multiple organs and was compared to the uptake at the pre-colonized and control implant sites, as well as their corresponding incisions [14]. Quantification was performed by manually drawing a volume of interest (VOI) in the SPECT-CT-fused scans, outlining the bladder, brain, heart, implant, incision site, liver, lungs, kidneys and the full body. The heart and lungs are in close proximity to each other; therefore, the VOI of the lungs was first drawn to include the heart, after which the VOI of the heart was subtracted. The uptake in the kidneys was measured by outlining the kidney on the side of the control implant only. Due to the high uptake and the position of the infected implant, spillover effects could wrongfully increase the measured uptake in the underlying kidney. The accumulation of ^111^In was defined as the percentage of the injected dose per cm^3^ (%ID/cm^3^), calculated as follows: (total activity in the organ or targeted area VOI (MBq)/(volume (cm^3^) × injected dose (MBq)) × 100%). The previously published data [14] were converted from the percentage of total body activity to %ID/cm^3^, as this allowed for a more accurate comparison. Additionally, the uptake at the infection site was compared to that of the blood pool (i.e., muscle tissue) by analyzing the infection-to-muscle contrast ratio. The VOI of the muscles in the upper thigh was used as a reference. Finally, the effective and biological half-lives of 4497-^111^In were determined. The effective decay of 4497-^111^In was quantified by measuring the total body activity at 0, 24, 72 and 120 h using software analyses of SPECT-CT images, whereas the total body activity immediately after injection at 0 h represented the injected dose. The biological half-life was calculated using the following formula:

$$T_{effective}=\frac{T_{biological}\times T_{physical}}{T_{biological}+T_{physical}}$$

### 3.4. Statistical Analysis

Statistical analyses were performed using the Statistical Package for the Social Sciences (IBM Corp. Released 2017. IBM SPSS Statistics for Windows, version 25.0. Armonk, NY, USA) in order to determine significant differences between the uptake in different organs and the area of interest using one-way ANOVA followed by Bonferroni’s post hoc test. A *p*-value of less than 0.05 was considered a significant difference between the organs and the target implant. The nonlinear regression analysis was performed using GraphPad Prism (GraphPad Software, GraphPad Prism for Windows, version 9.3.0, La Jolla, CA, USA).

## 4. Discussion

This study analyzed the biodistribution of In^111^-labeled 4497 IgG1 and its uptake at the target location, the infected implant. High uptake at the implant site at all the timepoints was accompanied by uptake in blood-rich organs, such as the heart, liver, lungs and kidneys, which is a typical biodistribution pattern for IgG in mice (Figure 1) [21,22]. However, the increased liver uptake observed could be due to hepatic elimination and not solely because of its status as a blood-rich organ. In this respect, renal elimination is less likely as mAbs are too large to be filtered by the kidneys [23]. High uptake in the heart was probably due to high activity in the blood, as similar uptake was seen in the aorta. In this regard, 4497-IgG1 showed high selectivity to the implant infection site over time with a favorable biodistribution pattern. However, due to a low elimination rate, prolonged exposure to high activity could pose a toxicity risk to healthy tissue if a long-lived radionuclide is considered for radioimmunotherapy. The specificity of 4497-^111^In localization in the infected implant was confirmed by using a non-infected implant as a control. In addition, in a previous study [14], the specificity of 4497-^111^In for *S. aureus* infection in vivo was established using a palivizumab-^111^In antibody to respiratory syncytial virus as an isotype matching control.

The rapid localization of the antibody to the infected implant is key for successful diagnostics and treatment. In this regard, the 4497 IgG1 antibody holds promise in a theranostic approach to deliver diagnostic and therapeutic radionuclides to the infection site. For example, antibodies labeled with ^111^In or positron-emitting radioisotopes such as zirconium-89 could be a powerful diagnostic tool for SPECT and positron emission tomography (PET) imaging, where potentially even low-grade infection could be detected with high specificity and sensitivity. When combined with therapeutic radionuclides, a potential theranostic treatment might be possible. In this regard, retention of the antibody over time is important for long-lived radionuclides to employ their effects to the fullest extent.

Due to the retention of 4497 IgG1 at the infection site, long-lived radioisotopes, such as Actinium-225 (^225^Ac) (half-life 10 days, 5.9 MeV), are particularly interesting for our application in prosthetic joint infections. During its decay, ^225^Ac emits four α-particles and is therefore lethal at lower activities when compared to radioisotopes that emit only one α-particle, such as ^213^Bi. Although a low dose (<10 kBq) of ^225^Ac did not have robust bactericidal properties compared to ^213^Bi, higher doses could potentially be as destructive to bacteria and biofilm as ^213^Bi.^17^ Alternatively, intermediate or high energy beta-emitting radioisotopes, such as Lutetium-177 or Rhenium-188, respectively, could be used, having the advantage of being readily available compared to alpha-emitting radionuclides as they are clinically used to treat prostate cancer and metastatic bone pain [24,25].

The long retention time may have been influenced by the slow elimination of the antibody, whereas the fast elimination of the radionuclides from healthy organs is important in order to minimize collateral damage. Diagnostics and treatment with radiation are always prone to safety concerns. Amongst others, bone marrow suppression is a feared complication. Due to high activity in the blood pool, bone marrow suppression might be expected. However, future toxicity studies need to confirm this.

The latest developments in infection imaging with radiopharmaceuticals have recently been highlighted [26]. In order to improve the theranostic approach even further, smaller vehicles can be used, such as small proteins, nanobodies, such as heavy chain (VHH), or other single domain antibodies or peptides [27,28]. In general, a smaller size leads to increased elimination of the potentially dangerous remaining unbound radioimmunoconjugates. Another advantage of smaller delivery molecules is increased penetration into tissue and presumably the biofilm [29]. Other advantages include high stability, increased expression, solubility, specificity and effective doses, as well reduced toxicity. Another approach to reduce the radiation dose to non-target tissues is the use of pre-targeting [30], where the antibody accumulates at the infection site, after which it is radiolabeled in vivo and most of the unbound antibody has cleared from the blood. For example, a patient with a periprosthetic joint infection, where the implant is colonized with bacteria and biofilm, could be diagnosed and treated with small targeting agents labeled with gamma- and alpha-emitters. When combined with a pre-targeting system, this could still be very effective and could minimize collateral damage.

Usually, biodistribution studies consist of administration of the targeting antibody in a relevant mouse model, followed by the harvesting organs or tissue samples at specific timepoints and measuring the radioactivity or subjecting them to more elaborate techniques, such as mass spectrometry. Measuring biodistribution using SPECT and calculating it using software is as accurate and requires fewer mice, as one mouse can be scanned at multiple timepoints. Another advantage is the easy identification of antibody accumulation anywhere in the body, and thus not being restricted to a predetermined focus on specific organs. For example, 4497 IgG1 targets both the dorsal knees and hips, although this targeting is not significant compared to that of other organs. This targeting is most likely due to lightly inflamed knee joints, which are commonly seen in young mice. Some concerns were expressed about the potential toxicity of long-lived alpha-emitters, such as ^225^Ac (with a physical half-life of 9.9 days) or ^227^Th (with a physical half-life of 18.7 days), in the radioimmunotherapy of cancer. However, phase 1/2 clinical trials with ^225^Ac- and ^227^Th-labeled antibodies have demonstrated acceptable safety profiles [31,32,33,34]. We anticipate that the RIT of infections with long-lived alpha-emitters will reveal safety profiles that are similar to cancer RIT safety profiles.

Future studies that investigate the efficacy of a theranostic approach in the treatment of (implant) infections should include preclinical studies using radioimmunotherapy, such as using alpha- or beta-emitting radionuclides in the same subcutaneous implant infection mouse model or performing such experiments in an orthotopic model with an infected metal implant. If successful, a clinical study can be conducted starting with a small number of participants focusing on evaluating toxicity and adverse events. Eventually, radioimmunotherapy has the potential to reduce the high mortality and morbidity rates associated with implant infections, either as a standalone treatment or as an adjuvant therapy combined with antibiotics, with or without surgery.

## 5. Conclusions

The results of the in vivo nuclear imaging and biodistribution analyses of the 4497-IgG1 antibody showed that it specifically targets *S. aureus* and/or its biofilm in vivo. However, its low elimination rate could pose a risk to healthy tissue. Nevertheless, this antibody isotope formulation holds promise as a drug delivery system for diagnostics and as a bactericidal agent when using radioactive isotopes that can potentially eradicate the biofilm in (peri)prosthetic joint infections. These results indicate the need for further development of a preclinical treatment study in order to establish therapeutic efficacy and thereby paves the way for subsequent clinical trials.

## Data Availability

The datasets generated and/or analyzed during the current study are not publicly available due to the size of the files but are available from the corresponding author on reasonable request.
